# A brief comment on vaccinations for opportunistic microorganisms

**DOI:** 10.1186/s13054-017-1684-3

**Published:** 2017-05-05

**Authors:** Ferhat Arslan, Haluk Vahaboğlu

**Affiliations:** Department of Infectious Diseases and Clinical Microbiology, Faculty of Medicine, Istanbul Medeniyet University, Istanbul, Turkey

We read with interest the manuscript by Rello et al. published in *Critical Care* [1]. The authors conducted a randomized, placebo-controlled vaccine study against *Pseudomonas aeruginos*a among intensive care unit (ICU) patients and concluded that vaccination against *P. aeruginosa* failed to protect the patients from infection with this microorganism. Interestingly, invasive *P. aeruginosa* infections were more common among the vaccinated population (vaccinated, 39/263; placebo, 6/92; risk ratio (RR) 1.169; 95% confidence interval (CI) 1.027–1.332; one-sided Fischer’s exact test, *p* = 0.042; Stata 13.1).

Vaccines have been used with great success against pathogenic microorganisms such as Measles virus, Variola virus, and *Corynebacterium diphtheriae*. In the past decade, vaccine development against opportunistic and environmental microorganisms has gained attention. *Streptococcus pneumonia*, a member of the human microbiome, has been a target for vaccine developers. From this study, we now understand that *P. aeruginosa*, a ubiquitous environmental microorganism, is another potential vaccine target.

A common feature of opportunistic microorganisms is that, under normal conditions, they do not cause infections. A disruption of the natural defense barriers allows opportunistic colonizers to gain access to otherwise sterile sites and trigger inflammation and damage. Eliminating one of the colonizers will almost never reduce the risk of developing infections from opportunistic pathogens. Once the defense mechanisms are compromised, a new colonizer would gain access to the site and cause inflammation. During the 1940s, in the pre-antibiotic era, native colonizers such as *S. pneumonia* and *Haemophilus influenzae* were the leading cause of nosocomial pneumonia [2]. Following the introduction of antibiotics in medical practice, *Staphylococcus aureus* emerged as the leading etiological agent during the 1960s [3]. Colonizers, and consequently the causative microorganisms, changed but the incidence of nosocomial pneumonia did not decrease. Following the widespread use of pneumococcal vaccine, similar consequences were observed [4]. This study once more indicates that vaccines against opportunistic colonizers are ineffective in preventing infections.

However, the predominant worry is not the failure of vaccines against opportunistic pathogens. Widespread use of vaccines against members of the human microbiome or the normally non-pathogenic environmental microorganisms has the potential to unpredictably change our microenvironment [5]. More importantly, administering large amounts of antigens parenterally to humans, despite no tangible benefits, is a major ethical issue.
