# Supplementary material for: A brief comment on vaccinations for opportunistic microorganisms
Source: Crit Care. 2017 May 5;21:100. doi: 10.1186/s13054-017-1684-3 (PMC5418844; doi:10.1186/s13054-017-1684-3)
Supplement: Related texts

# Reply: Authors’ response

**Authors:** Jordi Rello, Anton Klingler

We appreciate the interest by Aslan and Vahaboğlu in our manuscript. Unfortunately, we respectfully disagree with the assertion that invasive *P. aeruginosa* infections were more common in IC43-vaccinated compared to placebo-injected ventilated ICU patients in our phase II report of a new, recombinant protein (OprF/I)-based vaccine against this opportunistic pathogen. As presented in the article, there was no significant difference observed in confirmed infection rates when randomized vaccination groups and placebo were compared overall with the pre-planned statistical test (Fisher-Freeman-Halton) [6]. The post-hoc comparison of all merged IC43 vaccination groups together against placebo by Arslan and Vahaboğlu using Fisher's exact test in a one-sided manner is statistically highly disputable and we are convinced that the observed infection rates do not allow the conclusion that IC43 neither increases nor reduces the risk of invasive *P. aeruginosa* infections.

In addition, the presented phase II study investigated two doses and two formulations of the IC43 vaccine against *P. aeruginosa* in ventilated ICU patients for immunogenicity as primary, and safety and efficacy as secondary, outcome measures. Consequently, as emphasized in our discussion, the study was insufficiently powered for evaluation of efficacy in terms of *P. aeruginosa* infection rates and the study methodology likely had biased detection of infections [1].

We oppose Arslan and Vahaboğlu’s statement that administration of antigens is a major ethical issue, as therapeutic benefit/risk assessment is the key concept of clinical development and regulatory drug approval. In contrast to the authors’ statement, there is evidence that vaccination against opportunistic pathogens can be successful, as demonstrated by a recent meta-analysis on the effectiveness of a 23-valent pneumococcal vaccine in the elderly [7].

Years into the development of effective vaccines against nosocomial infections, *P. aeruginosa* continues to cause serious infections, particularly in the critically ill, immunocompromised, and those with burn wounds, with cystic fibrosis or contact lens-associated bacterial keratitis [8]. Our data provide a rationale that vaccination against nosocomial *P. aeruginosa* infection may improve clinical outcome in mechanically ventilated patients exposed to nosocomial infections and warrants further testing in a pivotal Phase II/III study.

Taken together, we strongly believe that considering the problem of nosocomial infections, the prominent role of *P. aeruginosa* in this setting, antibiotic resistance, an aging population and population dynamics with a predicted doubling in population, a vaccine against *P. aeruginosa* is urgently needed. Indeed, MDR *P. aeruginosa* is classified as a “serious threat” by the CDC [9], and innovations to develop adjunctive therapies, such as a vaccine, should be a research priority.
